# Role of fatty acids in *Bacillus* environmental adaptation

**DOI:** 10.3389/fmicb.2015.00813

**Published:** 2015-08-05

**Authors:** Sara E. Diomandé, Christophe Nguyen-The, Marie-Hélène Guinebretière, Véronique Broussolle, Julien Brillard

**Affiliations:** ^1^INRA, UMR408 Sécurité et Qualité des Produits d’Origine VégétaleAvignon, France; ^2^Université d’Avignon, UMR408 Sécurité et Qualité des Produits d’Origine VégétaleAvignon, France; ^3^UMR 1333 DGIMI, INRA, Université de MontpellierMontpellier, France

**Keywords:** *Bacillus* genus, adaptation, FAS II, FA degradation, exogenous FAs

## Abstract

The large bacterial genus *Bacillus* is widely distributed in the environment and is able to colonize highly diverse niches. Some *Bacillus* species harbor pathogenic characteristics. The fatty acid (FA) composition is among the essential criteria used to define *Bacillus* species. Some elements of the FA pattern composition are common to *Bacillus* species, whereas others are specific and can be categorized in relation to the ecological niches of the species. *Bacillus* species are able to modify their FA patterns to adapt to a wide range of environmental changes, including changes in the growth medium, temperature, food processing conditions, and pH. Like many other Gram-positive bacteria, *Bacillus* strains display a well-defined FA synthesis II system that is equilibrated with a FA degradation pathway and regulated to efficiently respond to the needs of the cell. Like endogenous FAs, exogenous FAs may positively or negatively affect the survival of *Bacillus* vegetative cells and the spore germination ability in a given environment. Some of these exogenous FAs may provide a powerful strategy for preserving food against contamination by the *Bacillus* pathogenic strains responsible for foodborne illness.

## Introduction

Most vital bacterial cell functions are attributed to the plasma membrane (Parsons and Rock, 2013), particularly its ability to form a permeable barrier, pump essential metabolites and macromolecules into the cell and prevent the entry of undesirable solutes from the external environment (Weber and De Bont, 1996; Ramos et al., 2001). To adapt to a wide range of environments, bacteria have the ability to control the biophysical properties of their membranes (Zhang and Rock, 2008; Murínová et al., 2014), including the membrane fluidity necessary for the growth and survival of bacteria in their environment (Esser and Souza, 1974; De Sarrau et al., 2012). Bacterial cell membranes are mainly composed of proteins, lipids and phospholipids (Bishop et al., 1967; Beaman et al., 1974). Glycerophospholipids (glycerol-based phospholipids) represent ∼90% of the macromolecules in bacteria (Neidhardt, 1996). They are mainly located on cell membranes and represent the main pool of FAs in microorganisms. In addition, FAs are important sources of metabolic energy and are important effector molecules that regulate metabolism.

The FA composition of bacterial cells varies depending on the species and has thus been used as a biomarker in taxonomy for many years (Cherniavskaia and Vasiurenko, 1983; Vasiurenko et al., 1984; Guinebretiere et al., 2013). Moreover, the FA composition of the cell membrane varies depending on environmental conditions because it plays a leading role in bacterial adaptation to environmental changes (Sinensky, 1971; Yano et al., 1998; De Sarrau et al., 2012). Exogenous FAs have been shown to influence the growth ability of *Bacillus* cells. For example, in *Bacillus cereus*, these exogenous FAs may impair (Lee et al., 2002) or improve growth (De Sarrau et al., 2013) depending on environmental conditions. In the last decade, the regulation of genes involved in metabolism and FA transport has been described in bacteria (Schujman et al., 2003; Pech-Canul et al., 2011), leading to the identification of regulators (Schujman et al., 2003; Dirusso and Black, 2004).

The genus *Bacillus* is the largest, most diverse and most prominent genus of aerobic endospore-forming bacteria (Fritze, 2004). In 2015, this genus contained ∼299 species and 7 subspecies (Euzeby, 2015). *Bacillus* strains are widely distributed in the environment, belong to different niches and include strains with economic and health interests. For instance, species of the genus *Bacillus* can have important roles in industrial processes (De La Fuente-Salcido et al., 2013; Liu et al., 2014; Pasvolsky et al., 2014), and some strains are also human pathogens (Lamanna and Jones, 1963; Bartoszewicz et al., 2013).

This review focuses on the nature of FAs in *Bacillus* and their role in adaptation to their close environment, addressing both the adaptation of species to their specific ecological niches and the adaptation of the bacterial cell to a changing environment. We describe and discuss the FA composition of *Bacillus* species by identifying common and specific elements of their FA pattern in relation to their ecological niches, thus examining the versatility of the FA pattern in relation to their biosynthesis during adaptation in various environments. Finally, we address the impact of exogenous FAs on the growth capacity of *Bacillus* species.

## Nature and Synthesis of the Main FAs in the *Bacillus* Genus

### Description of FA Profiles in the *Bacillus* Genus

**FIGURE 1:**
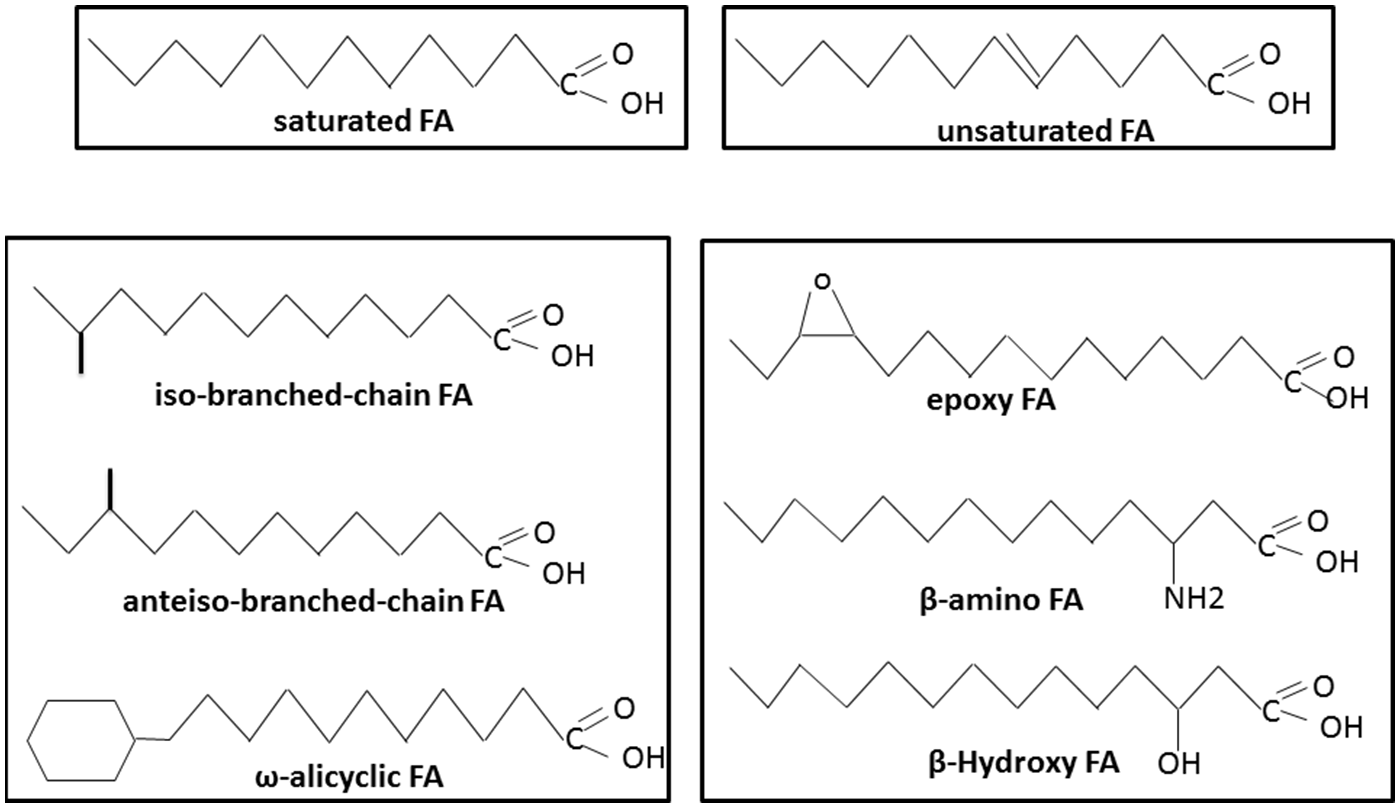
**Different types of fatty acids (FAs) present in *Bacillus* species**.

Like other Gram-positive bacteria, *Bacillus* species have three main groups of FAs: branched-chain FAs, straight-chain FAs, and complex FA types (such as cyclic, hydroxyl or epoxy FAs; Harris, 1996; **Figure 1**). Compared with other genera of Gram-positive bacteria, such as *Micrococcus, Clostridium*, and *Corynebacterium*, the genus *Bacillus* is characterized by a relative homogeneity of its FA composition across species (Moss and Lewis, 1967; Harris, 1996). To date, no *Bacillus* strains have been described with only branched-chain or straight-chain FAs. Another characteristic of this genus is that linear saturated FAs such as C_14:0_ or C_16:0_, which are encountered in the majority of microorganisms, are generally minor constituents in the genus *Bacillus* (Kaneda, 1977). *Bacillus* species are also characterized by displaying three major polar lipids: the phospholipids phosphatidylethanolamine, phosphatidylglycerol, and diphosphatidylglycerol (Bishop et al., 1967; Lang and Lundgren, 1970; Qiu et al., 2009; Zhai et al., 2012; Seiler et al., 2013; Yu et al., 2013; Choi and Cha, 2014; Jiang et al., 2014; Kosowski et al., 2014; Van Pham and Kim, 2014). However, some aminophospholipids are also found in the membranes of certain *Bacillus* strains (Bishop et al., 1967; Kang et al., 2013; Seiler et al., 2013; Wang et al., 2013; Choi and Cha, 2014).

#### Branched-Chain FAs

A singularity of the *Bacillus* genus is the abundance of branched-chain FAs (**Figure 1**; Kaneda, 1977; Kämpfer, 1994), with a predominance of branched chain iso and anteiso FAs containing 12–17 carbons (Berkeley et al., 2002). Branched-chain FAs also include ω-alicyclic FAs with or without modifications such as unsaturation and hydroxylation. Branched-chain FAs represent up to 98% of the total FAs depending on the species (Kaneda, 1969). More precisely, iso-C_15:0_, anteiso-C_15:0_, iso-C_16:0_, iso-C_17:0_, and anteiso-C_17:0_ represent the major FAs typically found in *Bacillus* species (Kämpfer, 1994; Song et al., 2000). Branched-chain FAs display a lower melting point temperature than their equivalent straight-chain FAs. Their presence in the membrane is therefore expected to increase its fluidity.

#### Unsaturated FAs

According to Kaneda, the proportion of unsaturated fatty acids (UFAs) varies in the genus *Bacillus* from 0 to 28% of the total FAs under optimal growth conditions (Kaneda, 1977). In *Bacillus* strains, UFAs consist almost exclusively of mono-unsaturated FAs at optimal growth temperature (Kaneda, 1977; **Figure 1**). Different systems of nomenclature are commonly used to describe UFAs. In the present review, we use the Δx (or delta-x) nomenclature, in which each double bond is indicated by Δx; the double bond is located on the xth carbon–carbon bond, counting from the carboxylic acid end of the molecule. The most common mono-unsaturated FAs generally encountered in living organisms are Δ9 isomers. However, most of the *Bacillus* strains also display Δ5, Δ8, and Δ10 isomers (Kaneda, 1977; Brillard et al., 2010; De Sarrau et al., 2012). As for the branched-chain FAs, UFAs display a lower melting point temperature than their equivalent saturated FAs and contribute to membrane fluidity.

#### Complex FAs

Several *Bacillus* species display unusual FAs. These complex FAs are generally hydroxy, amino, and epoxy FAs (**Figure 1**). β-hydroxy FAs or β-amino FAs, regardless of their saturation or branched structure, are linked to polypeptides and form surfactant and antimicrobial agents (Ongena and Jacques, 2008; Baindara et al., 2013; Mondol et al., 2013; Romano et al., 2013). Epoxy FAs, which consist of FAs with one or two epoxy group(s), can be produced by *Bacillus* genus species, and some possess antimicrobial properties (Celik et al., 2005; Hou, 2008). Unlike the branched and unsaturated FAs described above, these complex FAs have not been described as components of the *Bacillus* membrane.

### Major FAs and Phospholipid Biosynthesis in *Bacillus*

#### Saturated FA Biosynthesis

In living organisms, fatty acid synthesis (FAS) comprises a repeated cycle of reactions consisting of the condensation, reduction, dehydration, and subsequent reduction of carbon–carbon bonds. In higher eukaryotes, these reactions are performed by a large multifunctional protein forming the so-called FAS type I pathway (Chirala et al., 1997). FAS type II pathway reactions are catalyzed by discrete enzymes in bacteria, plant chloroplasts, and in the eukaryotic protozoan *Plasmodium falciparum* (Marrakchi et al., 2002; Freiberg et al., 2004; Van Schaijk et al., 2014). Two (or more) isoenzymes may be available to catalyze a given step of the pathway, although these enzymes may differ in substrate specificity and, hence, physiological function (Rock and Cronan, 1996). The set of genes that performs the reactions in the pathway are generally highly related and clearly identified among the *Bacillus* species.

**FIGURE 2:**
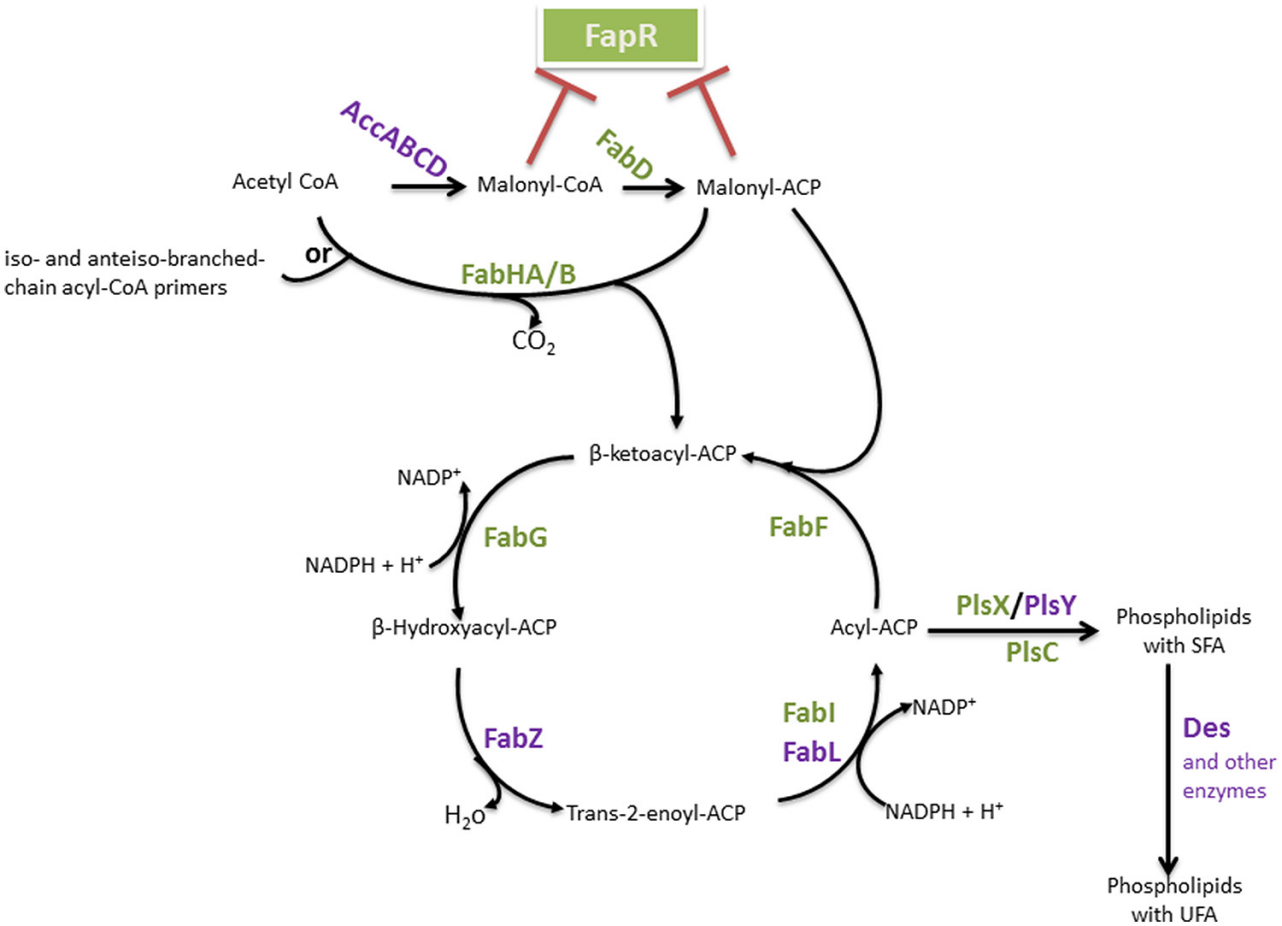
***Bacillus* FAS II pathway and FA integration into phospholipids.** The enzymes are identified in bold. The FapR major regulator of this pathway is represented, and enzymes which encoding genes belong to the *fap* regulon are indicated in green. Red blunt-head lines indicate a repression. AccABCD, acetyl-CoA carboxylase ABCD; FapR, fatty acid and phospholipid biosynthesis regulator; FabD, malonyl-CoA:ACP transacylase; FabH, β-ketoacyl-ACP synthase III, FabZ, β-hydroxyacyl-ACP dehydratase, FabI, enoyl-ACP reductase I; FabL, enoyl-ACP reductase III; FabF, β-ketoacyl-ACP synthase II; PlsX, acyl-acyl-ACP-phosphate acyltransferase; PlsY, acyl-phosphate- glycerol-phosphate acyltransferase; PlsC, 1-acylglycerol-3-P acyltransferase; Des, desaturase.

The biosynthesis of FAs is the first step in the formation of membrane lipids and represents a vital feature of bacterial physiology. This pathway has been extensively studied in Gram-negative *Escherichia coli* and Gram-positive *Bacillus subtilis*, which serve as models for understanding type II systems in other bacteria. The basic steps of the FAS cycle are common to all bacteria, and the genes encoding the enzymes are generally conserved (Marrakchi et al., 2002). We will describe the pathway that has been studied in *B subtilis* (Marrakchi et al., 2002; White et al., 2005; see **Figure 2**), highlighting differences in comparison to *E. coli*.

The first group of enzymes constitutes the initiation module (**Figure 2**). This group is a multisubunit enzyme that consists of acetyl coenzyme A (acetyl-CoA) carboxylase (ACC), which catalyzes a key step in intermediary metabolism that converts acetyl-CoA to malonyl-CoA. The overall ACC reaction requires four gene products: AccA, AccB, AccC, and AccD. Malonyl-CoA is transferred to the acyl-carrier-protein synthase enzyme (ACP) by the malonyl-CoA:ACP transacylase (FabD) to form malonyl-ACP (Marrakchi et al., 2002; White et al., 2005). FA synthesis is then initiated by the condensation of malonyl-ACP as the primer with acetyl-CoA as the acceptor.

This first condensation step is catalyzed by β-ketoacyl-ACP synthase III (FabH) to form β-ketobutyryl-ACP (a β-ketoacyl-ACP) and CO_2_. This process initiates a cycle of elongation of the acyl-ACP by two carbons units. A repetitive series of reactions occurs, with the addition of two-carbon units per cycle, until a final saturated FA is formed. The elongation module consists of protein condensation on an acyl-ACP primer with malonyl-ACP. The first reaction in this cycle is the NADPH-dependent reduction of β-ketoacyl-ACP to β-hydroxyacyl-ACP by β-ketoacyl-ACP reductase (FabG). The obtained β-hydroxyl intermediate is dehydrated to yield trans-2-enoyl-ACP, which is catalyzed by β-hydroxyacyl-ACP dehydratase (FabZ). The final step of the cycle is the NAD(P)H-dependent reduction of the double bond in the trans-2-enoyl-ACP intermediate by an enoyl-ACP reductase [enoyl-ACP reductase I (FabI), enoyl-ACP reductase III (FabL)] to form an acyl-ACP. β-ketoacyl-ACP synthase II (FabF) initiates the subsequent elongation cycles via the condensation of malonyl-ACP with acyl-ACP. Indeed, in contrast to *E. coli*, which expresses both FabF and FabB for the condensation steps, the sole condensation enzyme in *B. subtilis* for the subsequent elongation steps of FAS is FabF (Schujman et al., 2001).

To summarize, the three steps of FA biosynthesis are (i) initiation or synthesis of precursor molecules, (ii) condensation, and (iii) elongation cycles.

#### Branched-Chain FA Biosynthesis

*Bacillus subtilis* and all of the *Bacillus* genus species produce mainly branched-chain FAs. For the synthesis of this FA group, the same set of FAS enzymes is used. It has been shown that *B. subtilis* possesses two FabH isoenzymes, FabHA and FabHB, both of which preferentially utilize branched-chain acyl-CoA (**Figure 2**). Indeed, these enzymes carry out the initial condensation reaction of FA biosynthesis using acetyl-CoA as a primer, and they also utilize iso- and anteiso-branched-chain acyl-CoA primers as substrates, leading to the production of, primarily, iso- and anteiso-branched chain FAs (Choi et al., 2000). Acetyl-CoA is used as a substrate only for the synthesis of straight-chain FAs. Branched-chain FAs have been shown to arise from branched-chain amino acid (BCAA) metabolism (Willecke and Pardee, 1971; Kaneda, 1991), consisting of valine, leucine, and isoleucine. The metabolism of these amino acids produces short-chain branched acyl-CoAs, such as isobutyric-, isovaleric-, and 2-methylbutryric-CoA, respectively, which serve as primers for type II FA synthesis in place of acetyl-CoA (Willecke and Pardee, 1971; Choi et al., 2000; He and Reynolds, 2002). Isoleucine is the precursor of anteiso-branched chain FAs, while leucine and valine give rise to the primers for iso-branched FAs (Kaneda, 1977, 1991). The substrate specificity of the FabH-condensing enzyme is a determining factor in the biosynthesis of branched-chain FAs by FAS II (Choi et al., 2000); FabHA and FabHB demonstrate a slight preference for anteiso and iso precursors, respectively.

The branched-chain α-ketoacid decarboxylase, which has been shown to be essential for branched-chain FA biosynthesis, catalyzes the decarboxylation of α-ketoacids derived from BCAAs to generate branched-chain acyl-CoA primers (Willecke and Pardee, 1971; Lu et al., 2004). In *B. subtilis*, a mutation in the gene encoding this enzyme resulted in auxotrophy for branched-chain FA precursors derived from isoleucine, valine, and leucine (Willecke and Pardee, 1971; Boudreaux et al., 1981).

#### Unsaturated FA Biosynthesis

Fatty acid synthesis type II has been shown to be active in both aerobic and anaerobic UFA synthesis pathways in *E. coli* because it does not require molecular oxygen (Scheuerbrandt and Bloch, 1962; Cronan and Vagelos, 1972). However, in *Bacillus* species, *fabA* and *fabB* are lacking, which have been described in *E. coli* to be responsible for UFA biosynthesis. In all of the reported investigations, the biosynthesis of UFAs in *Bacillus* species requires oxygen (Beranova et al., 2010; De Sarrau et al., 2012).

*Bacillus subtilis* has been shown to have a single acyl-lipid oxygen-dependent desaturase, designated Des (Aguilar et al., 1998), that inserts a *cis*-double bond at the Δ5 position of the acyl chains of membrane phospholipids (Kaneda, 1972). A Δ5-desaturase has also been described in *B. megaterium* (Fulco, 1967) and *B. cereus* (Chazarreta Cifre et al., 2013). Ferredoxin and two flavodoxins (YkuN and YkuP) were identified to be redox partners of Δ5-desaturase because they act as electron donors in the desaturation reaction (Chazarreta-Cifre et al., 2011). This finding suggests that the three proteins might function physiologically in the biosynthesis of unsaturated FAs in *Bacillus* species (Chazarreta-Cifre et al., 2011). Some species, such as *B. cereus*, possess an additional Δ10-desaturase, which inserts a *cis*-double bond at the Δ10 position of the acyl chains of membrane phospholipids (Chazarreta Cifre et al., 2013).

#### Phospholipid Biosynthesis

Fatty acids mostly occurs in the form of phospholipids, and phosphatidic acid is the basic structure of the glycerophospholipids. Phosphatidic acid (PtdOH) is the biosynthetic product of the esterification of two FAs onto the two hydroxyl groups of glycerol-3-phosphate (G3P). G3P is a phosphate ester of the 3-carbon sugar glyceraldehyde, and the only known *de novo* pathway for the synthesis of this molecule in bacteria is the reduction of dihydroxyacetone phosphate by G3P synthase (GpsA; Kito and Pizer, 1969; Cronan and Bell, 1974; Ray and Cronan, 1987; Beijer et al., 1993; Morbidoni et al., 1995). PtdOH biosynthesis starts with the acylation of glycerol-3-phosphate (G3P) to form 1-acyl-G3P. Similarly to most Gram-positive bacteria, two enzyme systems carry out the first reaction in *B. subtilis*: PlsX (acyl-acyl-ACP-phosphate acyltransferase) and PlsY (acyl-phosphate-glycerol-phosphate acyltransferase) (**Figure 2**; Lu et al., 2007; Yoshimura et al., 2007). The soluble enzyme PlsX converts acyl-ACP to acyl-phosphate, and the membrane-associated PlsY transfers the acyl group from acyl-phosphate to glycerol 3-phosphate (Lu et al., 2007). Acylation of the 2-position of the 1-acyl-G3P is catalyzed by PlsC, a membrane-bound 1-acylglycerol-3-P acyltransferase that predominately uses acyl-ACP, although some PlsCs also use acyl-CoA. Indeed, FAs from the environment may be converted into acyl-CoA derivatives and incorporated into the bacterial membrane (Fulco, 1972; Krulwich et al., 1987; Lu et al., 2007).

### Regulation of FA Metabolism

**FIGURE 3:**
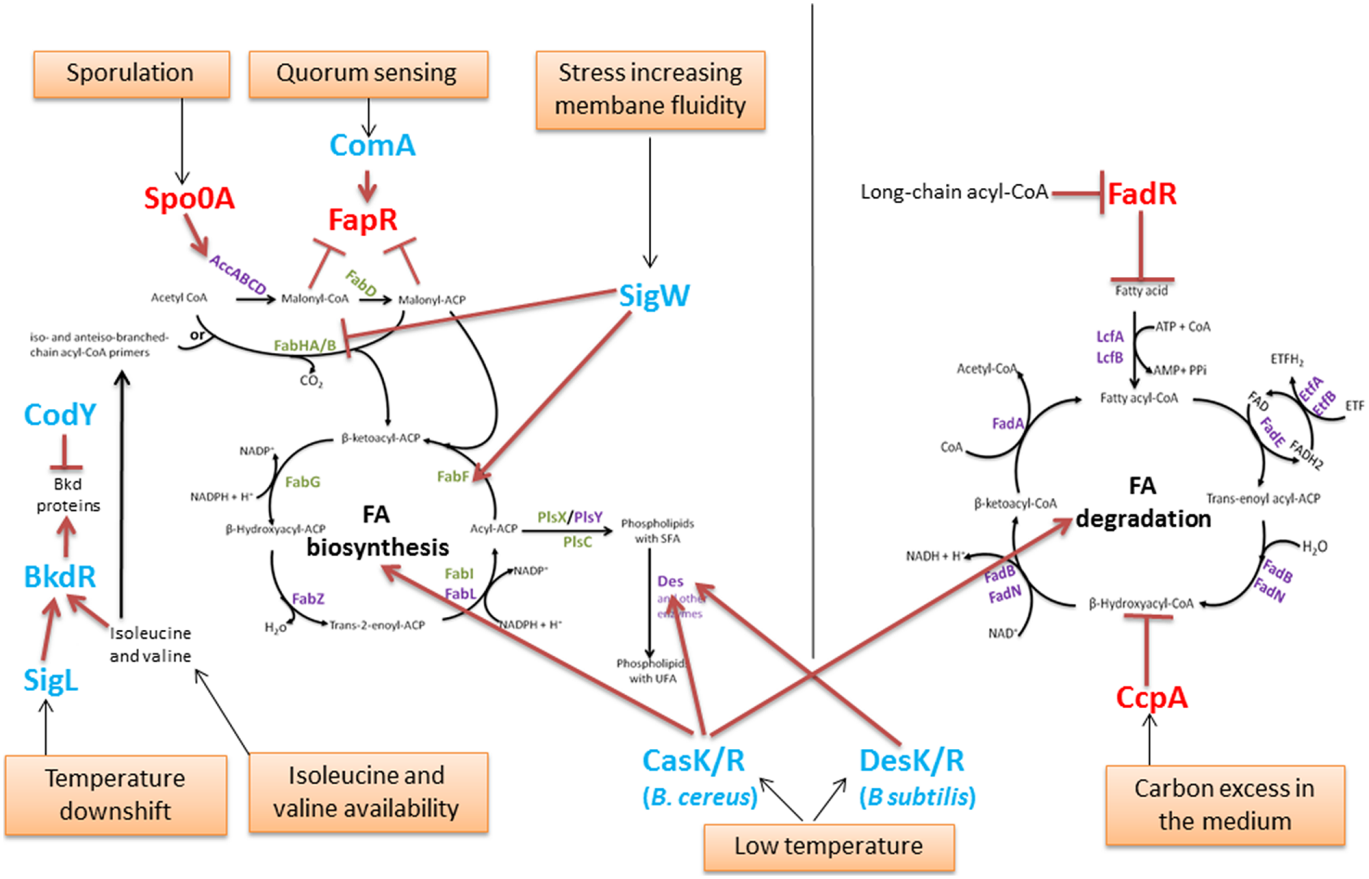
**Regulation of the FA pool in *Bacillus*.** The enzymes are identified in purple or in green (for those which encoding genes are regulated by the global regulator FapR, as in **Figure 2**). Global regulators of metabolism and sporulation are identified in red. Other regulators are in blue. Red blunt-head line and arrows indicate repression or activation, respectively. Orange boxes represent environmental conditions involved in FA regulation.

The FA pool results from FA biosynthesis, FA incorporation into membrane phospholipids and FA degradation. FA biosynthesis is an energetically expensive process, which explains why the rate of FA production is tightly regulated to ensure that the supply of membrane phospholipids corresponds exactly to the needs of the cell. Indeed, the inhibition of phospholipid synthesis has been shown to result in a rapid decrease in the rate of FA synthesis and in the accumulation of acylated-derivatives of ACP (Rock and Jackowski, 1982; Heath and Rock, 1996). During the optimal growth or responses to changes in the environment of *Bacillus*, the FA composition is regulated to maintain cell membrane homeostasis. Here, we will review some key regulators of FA metabolism (**Figure 3**). Most of these regulators have been described in *B. subtilis*.

#### Regulation of the FA Pool During Optimal *Bacillus* Metabolism and Sporulation

In *B. subtilis*, the expression of FAS II genes is tightly regulated by the transcriptional regulator FapR (**Figure 2**). FapR regulates many genes involved in FA and lipid synthesis (the *fap* regulon) but not those encoding the ACC multisubunit enzyme. The genes *fabHA-fabF, fapR-plsX-fabD-fabG, fabI, fabHB, yhfC* (putatively encoding a transmembrane protein likely involved in FA synthesis) and *plsC*, with *plsX* and *plsC* involved in phospholipid biosynthesis, belong to the *fap* regulon (Schujman et al., 2003; Fujita et al., 2007). FapR is highly conserved in many Gram-positive organisms, including all of the species in the *Bacillus, Listeria, Staphylococcus*, and *Clostridium* genera and other related genera (Schujman et al., 2003). FapR is repressed by the first two intermediates of FAS, malonyl-CoA and malonyl-ACP (**Figures 2** and **3**), to balance the production of membrane phospholipids and thus maintain membrane lipid homeostasis.

The degradation of FA via β-oxidation has a crucial function only under certain physiological conditions (Fujita et al., 2007), e.g., during sporulation (Gonzalez-Pastor et al., 2003) or calcium carbonate biomineralization (Barabesi et al., 2007). The *B. subtilis* FA degradation is regulated by FadR (**Figure 3**) from the TetR-family of transcriptional regulators, a homolog of FabR found in Gram-negative bacteria. FadR represses FA β-oxidation degradation by repressing five *fad* operons: *lcfA-fadR-fadB-etfB-etfA, lcfB, fadN-fadA-fadE, fadH-fadG*, and *fadF-acdA-rpoE*, (Matsuoka et al., 2007; Fujihashi et al., 2014). FadR binds to boxes located in the promoter regions of these operons. The FadR activity is inhibited by the long-chain acyl-CoAs (14–20 carbon molecules), leading to the induction of FA degradation (Matsuoka et al., 2007; Fujihashi et al., 2014).

The catabolic control protein CcpA represses many catabolic genes and operons, consisting mainly of those involved in carbon, nitrogen, and phosphate metabolism. It has also been described to repress the five operons belonging to the FadR regulon, including the *fadR* operon (Tojo et al., 2011).

Spo0A represents a master transcription factor in *Bacillus* strains because the Spo0A regulon comprises more than 500 genes (Piggot and Hilbert, 2004) and plays a major role in the formation of *Bacillus* spores. Active and robust *de novo* FA and membrane lipid synthesis occur during sporulation (Schujman et al., 1998; Pedrido et al., 2013). Spo0A controls the *accDA* operon involved in malonyl-CoA synthesis and, consequently, regulates FAS II by the negative modulation of FapR (Pedrido et al., 2013).

#### Examples of Regulations in the *Bacillus* FA Pool Based on Specific Responses

BkdR is a regulator that has been described in *B. subtilis*; it controls the utilization of isoleucine and valine as sole nitrogen sources (Debarbouille et al., 1999). Indeed, BkdR positively regulates the *bkd* operon, which consists of seven genes encoding enzymes that catalyze the conversion of BCAAs into precursors of branched FAs (**Figure 3**). The expression of the *bkd* operon has been shown to be induced by the presence of isoleucine or valine in the growth medium and to depend on the presence of the sigma factor SigL (Peng et al., 2015). In addition, it is induced by a temperature downshift from 37 to 18°C in *B. subtilis* (Nickel et al., 2004). SigL and BkdR have been shown to participate in *B. subtilis* cold-shock adaptation (Wiegeshoff et al., 2006).

Branched-chain amino acids comprise isoleucine, leucine, and valine. Their biosynthesis must be thoroughly controlled to ensure that appropriate amounts of precursors are available for the synthesis of branched-chain FAs and of proteins and coenzyme A (Brinsmade et al., 2010). CodY is a global transcriptional regulator that is widespread among *Bacillus* (Slack et al., 1995; Hsueh et al., 2008; Van Schaik et al., 2009), responsible for responding to these metabolites and regulating the genes that direct their synthesis (Brinsmade et al., 2010). Moreover, CodY negatively regulates the *bkd* operon involved in the degradation of isoleucine and valine (Debarbouille et al., 1999).

ComA, which belongs to the major quorum response Com signaling pathway in *B. subtilis*, was shown to regulate, both directly and indirectly, the expression of genes involved in FA metabolism, including FapR (Comella and Grossman, 2005). Indeed, the expression of the FapR regulon was shown to be reduced in a *comA* null mutant (Comella and Grossman, 2005). Because adjustment of the lipid composition of membranes is important for growth and gene regulation under a variety of different conditions, Comella and Grossman (2005) hypothesized that during the quorum response, changes in the lipid composition of the membrane may be important for accommodating the wide variety of developmental changes that might occur during competence, sporulation and biofilm formation.

The extracellular function (ECF) of SigW σ factor in *B. subtilis* is involved in the stress response to compounds that increase membrane fluidity (Kingston et al., 2011). The binding of SigW to the SigW-dependent promoter within the *fabHA-fabF* operon down-regulates *fabHA* encoding for the enzyme that initiates the synthesis of new FA chains (preferentially from branched precursors), but it also up-regulates *fabF* encoding the enzyme that initiates a new elongation cycle of the FA chain, leading to a higher proportion of straight chain FAs and a longer average chain length of the membrane phospholipids. These membrane alterations result in reduced bilayer fluidity and an increased resistance to detergents and antimicrobial compounds produced by other *Bacillus* species (Kingston et al., 2011).

Some two-component systems (TCSs) have been described regarding their role as thermosensors to maintain membrane homeostasis.

*Bacillus subtilis* DesK/R was the first TCS to be described for the maintenance of membrane fluidity during low-temperature adaptation in a *Bacillus* strain (Aguilar et al., 2001). DesK/R was shown to be effective either at constant cold temperatures or after a temperature downshift in response to sensing a decrease in membrane fluidity (Cybulski et al., 2002). The sensor protein DesK is a multipass transmembrane histidine kinase that senses an increase in membrane thickness when the growth of *B. subtilis* decreases (Aguilar et al., 2001; Cybulski et al., 2010; Inda et al., 2014). Its cognate response regulator DesR then regulates the expression of the Δ5-desaturase gene *des*, which is responsible for increasing the proportion of membrane UFAs and thus maintaining an optimal membrane fluidity (Aguilar et al., 2001).

CasK/R has been recently described as a TCS that is involved in *B. cereus* cold adaptation (Diomandé et al., 2014). In contrast to the membrane protein DesK, CasK presumably has a cytoplasmic location (Diomandé et al., 2014). Recent evidence has shown that this TCS regulates genes involved in both FA synthesis and FA degradation (Diomandé et al., 2015a), including two genes encoding the desaturases *desA* and *desB* in *B. cereus.* During growth at low temperatures, CasK/R regulates the membrane FA composition and, mainly, UFA synthesis by regulating *desA*. DesA is a Δ5-desaturase, homolog of the *B. subtilis* Des protein. Several studies identified in *Bacillus* species the Δ5 desaturase that is involved in adaptation at low temperatures (Fulco, 1967, 1969, 1972; Kaneda, 1972; Bredeston et al., 2011). The two examples of DesK/R and CasK/R illustrate that the regulation of membrane fluidity at low temperature via UFAs may involve different TCSs in *Bacillus* species.

In conclusion, in these two examples, the membrane fluidity appears to be maintained and, therefore, to adapt to the FA pool. *Bacillus* cells use several regulators that can act at four levels: precursor metabolism, FA and phospholipid synthesis, and FA degradation. Depending on the *Bacillus* environment, physical parameters such as temperature or stressful conditions influencing the membrane fluidity, but also chemical parameters such as nutrient availability, specific regulators are recruited for the adaptation and preservation of the membrane.

## FA Profiles Vary Among *Bacillus* Species

The *Bacillus* genus is a large genus in which the number of novel strains characterized is increasing (Choi and Cha, 2014; Kosowski et al., 2014; Zhao et al., 2014). The FA profiles of *Bacillus* genus strains are used, in addition to various other molecular data, as a biomarker for taxonomy.

The tools used for FA-based taxonomy have been standardized. For FA pattern analysis, the FAs were extracted in the form of FA methyl esters (FAMEs) using the MIDI method as previously described (Sasser, 1990; Connor et al., 2010; Guinebretiere et al., 2013). This MIDI method has been commonly used since the end of the 1990s to complement other standard discriminating tools and perform taxonomy, in particular for the *Bacillus* genus (Song et al., 2000). The MIDI method has also been used to describe new species of the genus (Reddy et al., 2008; Logan et al., 2009; Yu et al., 2013; Kosowski et al., 2014; Subhash et al., 2014).

Here, we discuss the extent to which the *Bacillus* FA patterns can discriminate species and their temperature adaptations.

### Kaneda’s Classification

#### Unsaturated FAs

**FIGURE 4:**
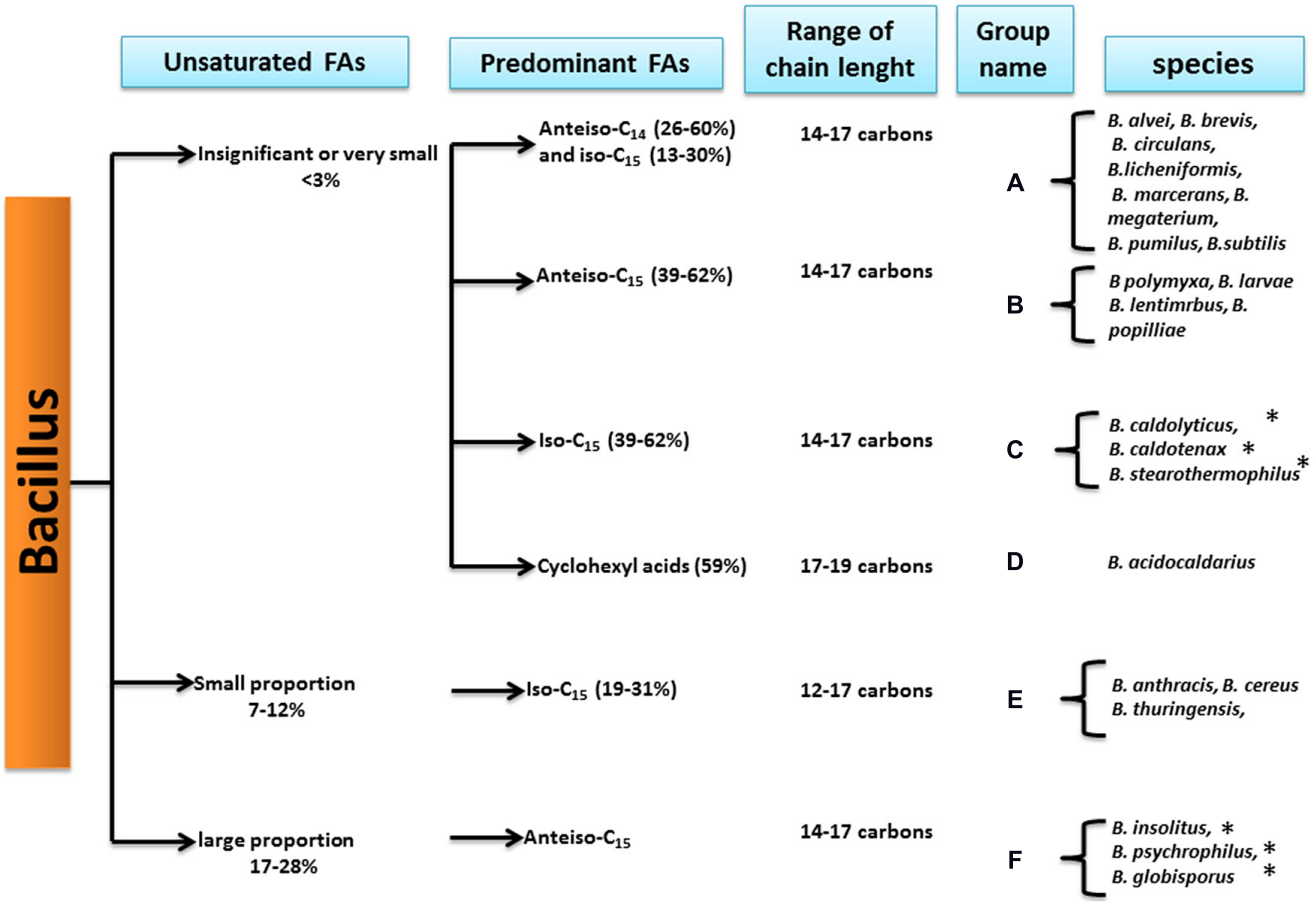
***Bacillus* species classification based on the fatty acid patterns (from Kaneda, 1977).** ^∗^Indicates that the species has been reclassified into another genus.

According to Kaneda, the *Bacillus* genus strains can first be divided into three groups depending on their UFA proportions (Kaneda, 1977; **Figure 4**).

First, species displaying a very low or insignificant proportion of UFAs, from 0 to less than 3% of the total FAs, formed the first group. This group included mesophilic and thermophilic strains, such as *B. subtilis, B. megaterium, B. stearothermophilus*, and *B. acidocaldarius* (see **Figure 4**). Further studies confirmed that at optimal temperatures, *B. subtilis* strains displayed nearly zero UFAs (Bishop et al., 1967; Grau and De Mendoza, 1993).

The second group consisted of strains displaying a small proportion of UFAs representing from 7 to 12% of the total FAs. This group included strains from the *B. cereus* group. In addition, some bacteria in this group, such as *B. weihenstephanensis* and *B. mycoides*, which are psychrotolerant strains of *B. cereus sensu lato*, were described after the Kaneda classification.

The third group consists only of psychrotolerant species of the genus, which have been described to present larger proportions of UFAs (17–28%). Studies of the three psychrotolerant species revealed that they displayed a larger proportion of UFA when compared to other strains, e.g., *B. globisporus* (26.1% of the total FAs), *B. insolitus* (25.1% of the total FAs), and *B. psychrophilus* (18.4% of total FAs) (Kaneda et al., 1983).

However, during this study, the growth conditions used for the studied strains were not standardized but varied according to the strains, while the actual MIDI method was used for the standardized growth conditions employed for taxonomy. Therefore, we were curious about the results when using the FA profile obtained using the MIDI method.

#### Predominant FAs

Based on the FA profiles, Kaneda further divided the *Bacillus* genus species into six groups (Kaneda, 1977), displaying specific predominant FAs combined with different levels of UFA proportions (**Figure 4**).

In this classification, psychrotolerant strains with high proportions of UFAs all belonged to group F with anteiso-C15:0 as the major FA. Studies investigating several psychrotrophic strains from *B. globisporus, B. psychrophilus, B. insolitus* (Kaneda et al., 1983), and *B. pasteurii* (Yoon et al., 2001) confirmed that the predominant FA was anteiso-C15:0. Even if some of these species now belong to another genus, the *Sporosarcina* genus, the criterion of anteiso-C15:0 as one of the major FA was used as one of the references to describe new psychrotolerant species of *Bacillus* [i.e., *B. beringensis*, which displayed anteiso-C15:0 and iso-C15:0 as major FAs (Yu et al., 2011)].

Strains of *B. cereus sensu lato*, which includes *B. cereus sensu stricto, B. thuringiensis*, and *B. anthracis*, are characterized by small proportions of UFAs in Kaneda’s scheme and contain iso-C15:0 as a major FA (Song et al., 2000), representing group E (**Figure 4**). Further studies strengthened this observation for the *B. cereus sensu stricto* strains (Brillard et al., 2010; De Sarrau et al., 2012). Moreover, a novel thermophilic species, *B. cytotoxicus*, belonging to *B. cereus sensu lato*, was described for possess iso-C15:0 as the major FA (Guinebretiere et al., 2013; Diomandé et al., 2015b). This finding is in agreement with the Kaneda grouping of *B. cereus sensu lato* species in group E (**Figure 4**).

Species with very small proportions of UFAs were subdivided by Kaneda into four subgroups according to their predominant FAs:

– Group C (**Figure 4**) contains thermophilic species such as *B. stearothermophilus* (since reclassified as *Geobacillus stearothermophilus*), in which the major FA is branched-chain iso-C15:0 (Cho and Salton, 1966; Yao et al., 1970). A thermotolerant species, *B. cytotoxicus*, has also been shown to display the branched-chain iso-C15:0 as the predominant FA (Guinebretiere et al., 2013). *B. cytotoxicus* belongs to *B. cereus sensu lato*, and in this respect, it is more closely related to the Kaneda group E. Nevertheless, it has a much lower proportion of UFAs than other species of *B. cereus sensu lato* (Diomandé et al., 2015b), which is consistent with the definition of Kaneda group C.
– Group A contains the mesophilic *B. subtilis* and *B*. *megaterium*, and studies have shown that iso-C15:0 is the major FA (Kämpfer, 1994; Cybulski et al., 2002). In contrast to the Kaneda analysis, the other predominant FA is not anteiso-C14:0 but anteiso-C15:0.
– Group B contains the cold-tolerant *B. polymyxa* (Guinebretiere et al., 2001; now reclassified as *Paenibacillus polymyxa*), which contains a particularly high proportion of anteiso C15:0 FAs (**Figure 4**).
– Group D corresponds to *Bacillus* species in which the predominant FAs are cyclohexyl FA (**Figure 4**), also called ω-alicyclic FA (**Figure 1**). These *Bacillus* species have since been reclassified in the *Alicyclobacillus* genus and are characterized by both acidophilic and thermotolerant/thermophilic behavior types (Chan et al., 1971; Da Costa et al., 2009).

Thus, the classification according to the ratio of UFAs and predominant FAs proposed by Kaneda (1977) appeared to fit with both the taxonomic position and adaptation to temperature. However, the FA analytical tools evolved, and there are several exceptions to the Kaneda classification. For example, the predominant FAs in *B. cerembensis*, which has been described as a psychrotolerant species, appeared to be iso-C15:0 and iso-C16:1 (Reddy et al., 2008) and not anteiso-C15:0. Similarly, few *Alicyclobacillus* species are acidophilic and thermotolerant without possessing ω-alicyclic FAs (Da Costa et al., 2009).

Not all of these observations appear to be specific to the *Bacillus* genus. In the *Clostridium* genus, the mesophilic and psychrophilic strains have been characterized by a higher percentage of UFAs compared with the thermophilic strains (Chan et al., 1971), and some thermophilic Clostridia species from different thermal niches have been shown to display different predominant FA types (Chan et al., 1971).

### Discrimination of *Bacillus* Thermotypes by FAs

#### The Specific Case of *Bacillus cereus sensu lato*

*Bacillus cereus sensu lato* is a specific group of *Bacillus* strains that consists of seven phylogenetic groups characterized by different ranges of growth temperatures (Guinebretiere et al., 2008), exhibiting four major thermotypes: thermotolerant (group VII), mesophilic (groups I, III, and IV), mesophilic-psychrotolerant intermediary (group V), and psychrotolerant (groups II and VI), as shown in **Table 1**. In contrast to the other *Bacillus* strains, *B. cereus sl* strains display a significant amount of FAs with short chains of 12 and 13 carbons (more than 10% of the total FAs) and a smaller amount of anteiso-C_15:0_ (Kämpfer, 1994; Song et al., 2000; Haque and Russell, 2004).

**Table 1:** Various thermotypes of *Bacillus cereus sensu lato* and mean values of i16:1(5) proportion, i15/i13 ratio, HAI, a15/i15 ratio for each phylogenetic group.

Thermotype	Growth range T°C^1^	Phylogenetic groups^1^	i16:1(5) %^2^	i15/i13^2^	a15/i15	HAI
Thermotolerant	20–50°C	VII	0.02 ± 0.01	7.13 ± 1.70	0.26 ± 0.03	5.73 ± 0.82
Highly mesophilic	15–45°C	III	0.31 ± 0.02	1.57 ± 0.04	0.20 ± 0.08	3.62 ± 0.48
Mesophilic	10–45°C	IV	0.50 ± 0.03	1.14 ± 0.10	0.31 ± 0.03	3.61 ± 0.11
Mesophilic	10–43°C	I	0.05 ± 0.01	1.09 ± 0.04	0.27 ± 0.00	1.89 ± 0.10
Mesophilic-Psychrotolerant Intermediary	10–40°C	V	0.66 ± 0.06	0.91 ± 0.13	0.35 ± 0.07	4.46 ± 0.78
Psychrotolerant	7–40°C	II	1.24 ± 0.05	1.12 ± 0.12	0.35 ± 0.08	5.19 ± 0.84
Psychrotolerant	5–37°C	VI	1.09 ± 0.13	0.61 ± 0.13	0.40 ± 0.12	3.27 ± 0.84

*^1^Guinebretiere et al. (2008), ^2^Diomandé et al. (2015b).*

A previous study has shown that *B. cereus sl* strains display three specific predominant FAs: iso-C13:0 (i13), iso-C15:0 (i15) and C16:0 (n16), and a significant proportion of UFAs (Diomandé et al., 2015b). This study showed that two parameters discriminate the strains from the various phylogenetic groups of *B. cereus sl*: the i15/i13 ratio and the C16:1 (5) proportion (**Table 1**). The i15/i13 ratio discriminated the major thermotypes of *B. cereus sl* and tended to decrease with the psychrotolerance. Indeed, this ratio was high (i.e., >1, up to 7) in the thermotolerant strains of group VII, exhibiting a value of ∼1 for mesophilic and intermediary strains and a low value (i.e., <1) for the psychrotolerant strains in group VI. The C16:1(5) proportion was more discriminating than the i15/i13 ratio because this proportion even differed between phylogenetic groups in close growth temperature ranges and tended to decrease with the thermotolerance (Diomandé et al., 2015b).

Thus, these data suggest that based on the FA predominance and key FA ratios, it is possible to predict the thermotype of strains of *B. cereus sl.* Even if the results of this study strengthen some of the observations reported by Kaneda, they also highlight the key role of specific FAs (particularly iso-C13:0) in the classification of *B. cereus sl*. These differences in FA composition among different thermotypes of *B. cereus* retrospectively also explain the results published by Pirttijärvi et al. (1998, 2000). These authors found that the strains isolated from processed whey and paper materials displayed FA profiles that were very different from those of the reference *B. cereus* strains used in the MIDI database; in the case of whey, the results were very different from those of the strains isolated from raw milk. In addition, the strains obtained from the processed whey were unable to grow at cold temperatures, in contrast to the strains from raw milk (Pirttijärvi et al., 1998). These processes presumably selected strains from the most heat-tolerant thermotypes of *B. cereus*, which present peculiar differences in FA composition compared with cold-tolerant thermotypes, as described above.

#### *Bacillus* and Related Genera excluding *B. cereus sensu lato*

##### Use of FA profiles data for discrimination of *Bacillus*

In the literature relating FA profiles, two criteria are described to discriminate species of the genus *Bacillus*: (i) the heat adaptation index (HAI; Connor et al., 2010) which is based on temperature adaptation factors and (ii) the a15/i15 ratio (Kämpfer, 1994) which is based on the predominant FAs (FAs with the largest proportion). The i15/i13 ratio is optimized for *B. cereus sl* strains which possess an important proportion of i13:0 compared with a15:0, as outlined in the previous section. However, neither HAI nor a15/i15 is relevant for discriminating thermotypes of *B. cereus sl* (**Table 1**). Thus, this section deals with *Bacillus* and relatives, excluding *B. cereus sl*, and are mentioned as “*Bacillus es”* in the following text, designing ‘*Bacillus* in every sense.’

###### Heat adaptation index

Connor et al. (2010) revealed that some FAs allow the discrimination of *B. subtilis* and *B. licheniformis* species that belong to different ecotypes by calculating the HAI. To determine this parameter, two factors must be calculated: (i) the high temperature adaptation factor as the sum of the proportions of the n14:0; n16:0; i14:0; i15:0; i16:0 and i17:0 FAs, (ii) the low-temperature adaptation factor as the sum of the proportions of a15:0; a17:0; n16:1; i17:1 (n-10) and 16:1 ω7c alcohol. The HAI is obtained by dividing the high temperature adaptation factor by the low temperature adaptation factor (Connor et al., 2010).

$$HAI=\frac{\begin{array}{c} p(n14:0)+p(n16:0)+p(i14:0)+p(i15:0)+p(i16:0)+p(i17:0) \end{array}}{\begin{array}{c} p(a15:0)+p(a17:0)+p(n16:1)\;+p(i17:1\;(n-10))+p(16:1\omega \;7c\;\mathrm{alcohol}) \end{array}}$$

p is the proportion of the FA.

###### a15/i15 ratio

The a15/i15 ratio (Kämpfer, 1994) exploits the two predominant FAs i15:0 and a15:0 in *Bacillus*, with a15:0 being the major FA in psychrotolerant strains (Kaneda, 1977).

As to our knowledge it doesn’t exist any study comparing these two criteria, we wondered which one was the more appropriate to discriminate species of different thermotypes among *Bacillus es* by using the FA profile. We therefore choose to compare these two criteria using the *Bacillus es* FA pattern data obtained from MIDI extraction by (Song et al., 2000; see **Table 2** for species of concern). These data, from the year 2000, include species that were later characterized to belong to other genera, e.g., *Sporosarcina, Paenibacillus, Geobacillus*, and *Lysinibacillus* (Pettersson et al., 1999; Nazina et al., 2001; Yoon et al., 2001; Ahmed et al., 2007).

**Table 2:** Strains (from Song et al., 2000) used for *Bacillus* genus classification using FA composition, respective reviewed name and growth temperature range obtained from the Bergey’s manual (Vos et al., 2011) and the database ABIS encyclopedia (Abis [ABIS v.9], n.d.).

Strains	Growth temperature range
*Bacillus subtilis170311*	ND
*B. aminovorans 94021 T*	5–10 to 37 °C
*B. amyloliquefaciens 170517*	15 to 50°C
*B. amyloliquefaciens 170518*	16 to 50°C
*B. amyloliquefaciens 94022*	17 to 50°C
*B. atrophaeus 95007 T*	10 to 55°C
*B. atrophaeus ATCC 9372*	10 to 55°C
*B. badius 94024*	15 to 50°C
*B. circulans CCTCCAB 94026*	5–20 to 35–50°C
*B. coagulans CCTCCAB 92066*	15–25 to 55–60°C
*B. firmus CCTCCAB 170519*	5–20 to 40–50°C
*B. firmus CCTCCAB 94028 T*	5–20 to 40–50 °C
*B. globisporus CCTCCAB 94031 T (Sporosarcina globispora)*	0–3 to 25–30°C
*B. insolitus CCTCCAB 94032 T (Psychrobacillus insolitus)*	0–5 to 25°C
*B. lentimorbus CCTCCAB 94034 T* (*Paenibacillus lentimorbus)*	20 to 35°C
*B. lentus CCTCCAB 94035 T*	10 to 40°C
*B. licheniformis CCTCCAB 170513*	15 to 50–55°C
*B. licheniformis CCTCCAB 170514*	16 to 50–55°C
*B. licheniformis CCTCCAB 94036 T*	17 to 50–55°C
*B. megaterium 170201*	3–20 to 35–45°C
*B. megaterium 170505*	3–20 to 35–45°C
*B. megaterium CCTCC AB 92075 T*	3–20 to 35–45°C
*B. mojavensis CCTCC AB 96001 T*	10 to 55°C
*B. niacini CCTCC AB 95011 T*	10 to 40°C
*B. pumilus CCTCC AB 94044 T*	5–15 to 40–50°C
*B. simplex CCTCC AB 94045 T*	20 to 40°C
*B. sphaericus 170511 (Lysinibacillus sphaericus)*	10–15 to 30–45°C
*B. sphaericus 170512 (L. sphaericus)*	10–15 to 30–45°C
*B. sphaericus CCTCC AB 92073 T (L. sphaericus)*	10–15 to 30–45°C
*B. stearothermophilus CCTCC AB 92070 T (Geobacillus stearothermophilus)*	30–45 to 65–75°C
*B. subtilis CCTCCAB 92068 T*	5–15 to 40–45 °C
*B. subtilis 170221*	5–15 to 40–45°C
*B. subtilis 170312*	5–15 to 40–45°C
*B. thiaminolyticus CCTCC AB 95017 T (P. thiaminolyticus)*	20 to 45°C
*Brevibacillus agri CCTCC AB 95005 T*	5–20 to 40°C
*Brevibacillus brevis CCTCC AB 94025 T*	10–35 to 40–50 °C
*P. amylolyticus CCTCC AB 95019 T*	10–15 to 40°C
*P. larvae CCTCC AB 94033 T*	20 to 40°C
*P. pabuli CCTCC AB 95012 T*	5–10 to 35–40°C
*P. peoriae CCTCC AB 95013 T*	5–10 to 35–45°C
*P. polymyxa 170507*	5–10 to 35–45°C
*P. polymyxa 170508*	5–10 to 35–45°C
*P. polymyxa CCTCC AB 92076 T*	5–10 to 35–45°C

##### Evaluation of the two ratios HAI and 15/i15 as indicator of species thermotype

**FIGURE 5:**
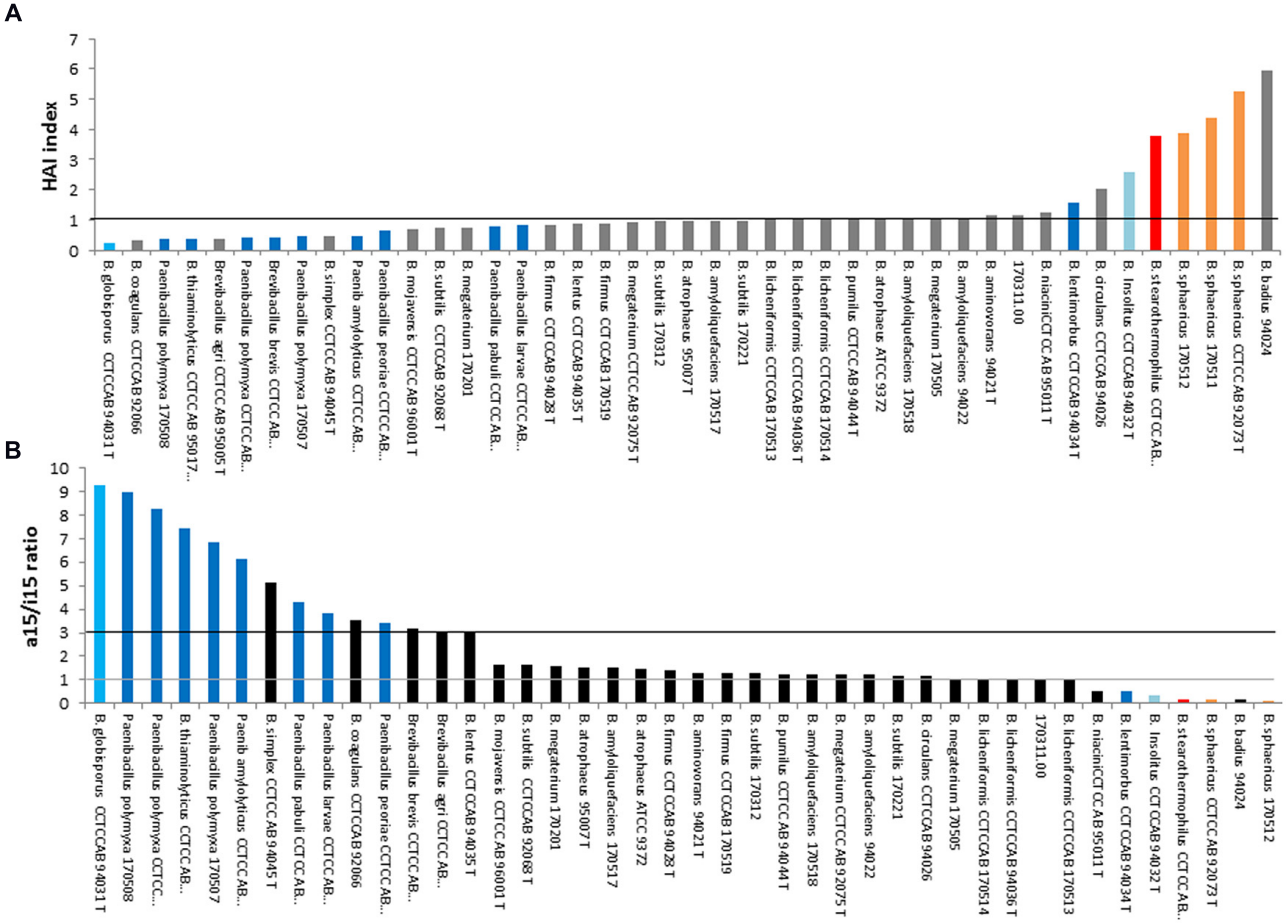
**Heat adaptation index **(A)** and a15/i15 ratio **(B)** among strains of the genus *Bacillus*, excluding *Bacillus cereus sensu lato*.** The strains displaying values close to the black line **(A)** or between the gray line and the black line **(B)** are mesophilic strains. Reclassified species are: *Sporosarcina* (blue), *Peanibacillus* (dark blue), *Geobacillus* (red), *Lysinibacillus* (orange), *Psychrobacillus* (pale blue). Gray **(A)** or black **(B)**: *Bacillus* strains.

Heat adaptation index and a15/i15 ratios are presented in **Figures 5A,B** respectively. Their interest for describing species thermotypes was examined below. Adaptation to temperature may have occurred by extending growth limits toward low or high temperature, resulting to different thermotypes. The most common are “psychrophilic,” “mesophilic” and “thermophilic” thermotypes. However, thermotypes also includes intermediate organisms, such as mesophilic strains with psychrotophic or with thermotolerant abilities.

Firstly, the psychrophylic species *Sporosarcina globispora* [previously named *Bacillus globisporus* (Yoon et al., 2001)] displayed the lowest HAI (**Figure 5A**) and highest a15/i15 ratio (**Figure 5B**). Psychrotrophic species of the genus *Paenibacillus* also appeared to display the same pattern in both representations: a low HAI (<1) and a high a15/i15 ratio (>3). Thus, this pattern seemed to overall describe most species able to grow at cold temperatures (from psychrophilic species to mesophilic species with psychrotrophic abilities).

Secondly, 22 strains that displayed values between 1 and 3 for a15/i15 and ∼1 for HAI, presumably belong to mesophilic species (*B. lentus, B firmus, B. circulans, B. aminovorans, B. subtilis* and closely related species *B. amyloliquefaciens, B. atrophaeus, B. mojavensis, B. licheniformis* and *B. pumilus*). This pattern was thus considered as specific of most mesophilic species not able to grow at cold temperatures (i.e., not psychrotrophic).

Thirdly, the thermophilic *Geobacillus sterarothermophilus* [previously known as *B. sterarothermophilus* (Nazina et al., 2001)] and the thermotolerant *B. badius* displayed a high HAI (**Figure 5A**) and a low a15/ i15 ratio (**Figure 5B**). This pattern was thus considered as indicating most of highly thermotolerant species.

##### Limitations of such index use

In this study, there were a number of exceptions for which the previous patterns were not followed. The psychrophilic *B. insolitus* [now *Psychrobacillus insolitus* (Krishnamurthi et al., 2010)] did not follow the psychrotolerant-specific pattern (i.e., low HAI and high a15/i15 ratio); the mesophilic *B. sphaericus* and *B. lentimorbus* harbored entirely or partly a thermotolerant-specific pattern; the thermotolerant to mesophilic *B. coagulans* and *Brevibacillus* harbored a psychrotolerant-specific pattern.

Different reasons may explain these exceptions:

(i) Intraspecies variability often taken into account to determine range of growth temperature (i.e., known thermotype) may be not truly represented by the reference strains used in this study.
(ii) In some cases the intraspecific variability may be due to bacterial complex containing different genomospecies. Indeed, numerous *Bacillus* complexes have been rearranged since years 2000, such as *B. sphaericus, B. coagulans, B. circulans, Br. brevis* complexes; these rearrangements generated new *Bacillus* species and new genus (Heyndrickx et al., 1995; Shida et al., 1997; Pettersson et al., 1999; Uetanabaro et al., 2003; Vos et al., 2011). Some of these genera are represented in **Figure 5** (*Paenibacillus, Sporosarcina, Geobacillus, Lysinibacillus, Psychrobacillus).* This could have contributed to separate entities with different thermotypes. For example, *Paenibacillus* genus originating from mesophilic *Bacillus* is known to contain mesophilic species mostly characterized by psychrotrophic abilities (Ivy et al., 2012). The data used here come from a study performed before most of these rearrangements, thus some may contain incoherencies.
(iii) As for *B. cereus sl* strains, some species (e.g., *B. coagulans* or *B. sphaericus*) may display a FA profile with major FAs different compared to the other *Bacillus* strains (with i17:0/a17:0 or i16/i16 among major FAs) and were thus difficult to be characterized by a15/i15 ratio and HAI index.

Regardless of the method used, it appears important that the group of strain studied displays at least the same type of dominant FAs (branched-chain C15 FAs) to avoid a biased analysis. Indeed, the FA profiles of some *Bacillus* species or related genera are so different that it can lead to the inappropriate determination of thermotype for the strains (i.e., *B. cereus sl, B. coagulans, Lysinibacillus, Psychrobacillus*).

The observation that a15:0 and i15:0 are also present in HAI and that the two methods resulted in the same global results may indicate that the other FAs utilized in HAI have a minor role in the thermotype determination.

#### Perspectives in this Field

In conclusion, the a15/i15 ratio for several *Bacillus* and related genera and the i15/i13 ratio and C16:1(5) proportion for *B. cereus sl* species may be good indicators of temperature adaptation but need to be further evaluated. Since 2010, taxonomists imposed the description of the FA profile for all newly described species. More accurate data on a wider range of well delimited species and representative strains of *Bacillus* should strengthen the proposed link between the FA profile of a strain/species and its thermotype.

## Changes in FA Composition During *Bacillus* Adaptation

### Changes Depending on Food Components and the Growth Medium Composition

The growth medium (including food) may also influence the FA composition of several strains of the *Bacillus* genus. For instance, UFAs from spinach or from a growth medium supplemented with lecithin have been detected in the membrane FAs of *B. cereus* (De Sarrau et al., 2013), leading to an increase in membrane fluidity, improved growth at low temperatures and anaerobiosis.

Strains of *B. cereus* isolated from rice were also tested for their growth ability at reduced temperatures and reduced water activity in rice starch (Haque and Russell, 2004). Rice starch stimulated the growth of the tested strains and modified the FA composition by increasing the proportion of branched-chain FAs and the ratio of iso/anteiso. In contrast to spinach (De Sarrau et al., 2013), UFAs from rice were not detected in *B. cereus* FAs (Haque and Russell, 2004).

When the growth of *B. thuringiensis* was performed in the presence of compounds that are specific precursors for branched-chain FA biosynthesis, such as butyrate, isobutyrate, valerate, and isovalerate, an increase in branched-chain FAs was observed in spores (Nickerson and Bulla, 1980).

In *B. subtilis*, the presence of isoleucine versus leucine, or of the respectively derived anteiso branched-chain FA precursors in the growth medium, increased the proportion of anteiso versus iso branched-chain FAs (Cybulski et al., 2002). In addition, the presence of isoleucine reduced the proportion of UFAs.

Ehrhardt et al. (2010) found that the FA composition of *B. cereus* spores was specific to the growth medium, with important variations in the proportions of i13, i15, n16, and C18:1 FAs and of total branched FAs.

### Changes Depending on the Temperature

The effect of temperature on the FA composition of *Bacillus* has been studied for years (Fulco, 1967). When confronted with a change in temperature, the species in the *Bacillus* genus modify their FA composition (Aguilar et al., 2001; Haque and Russell, 2004). These changes often involve branched-chain and unsaturated FAs.

When the temperature was raised to 60, 70, or 80°C, an increase in the iso-branched-chain FA iso-C17 and the linear C16:0 FA was observed in thermophilic species such as *B. caldolyticus* and *B. caldotenax* (Weerkamp and Heinen, 1972). In *B. simplex* strains grown at a high temperature (40°C), the iso-branched FA proportion increased, whereas the anteiso-branched and unsaturated FA proportions decreased (Sikorski et al., 2008).

Decreasing the growth temperature of some *Bacillus* genus species induced an increase in monounsaturated FAs and a decrease in saturated straight chain FAs. In addition to these changes, the proportion of branched-chain FAs (whether iso or anteiso-branched-chain FAs) increased with a decreasing temperature (Freese et al., 2008).

Similarly, an increased proportion of unsaturated FAs, but only of anteiso-branched-chain FAs, was observed in *B. simplex* strains isolated from specific habitats of a Canyon in Israel when grown at a low temperature (20°C) (Sikorski et al., 2008). A significant decrease in the relative concentration of all of the unbranched or iso-branched FAs was also observed. Reciprocally, at a high temperature (40°C), the proportion of iso-branched FAs increased, whereas the proportions of anteiso-branched and unsaturated FAs decreased (Sikorski et al., 2008).

Studies investigating *B. subtilis* and *B. cereus* adaptation to low temperature have underscored the key role played by UFAs (Aguilar et al., 2001) in the homeostasis of membrane fluidity at reduced temperatures (Haque and Russell, 2004; Brillard et al., 2010; Chazarreta Cifre et al., 2013). UFAs produced by Δ5-desaturase activity are specifically important for low temperature adaptation (Kaneda, 1972; Lombardi and Fulco, 1980; Mansilla et al., 2003). Some species such as *B. cereus* also possess a Δ10 desaturase that inserts a *cis*-double bond at the Δ10 position of the acyl chains of membrane phospholipids but is active regardless of the growth temperature (Chazarreta Cifre et al., 2013).

Some species in the *Bacillus* genus are facultative anaerobes (Rosenfeld et al., 2005; Yu et al., 2013; Tang et al., 2014). Under anaerobic conditions, *B. subtilis* and *B. cereus*, and presumably other *Bacillus* species that are devoid of FabA and FabB, are no longer able to synthesize UFAs. Thus, in the absence of oxygen (anaerobic respiration of nitrates), conditions in which FA desaturation by *B. subtilis* is not possible, the anteiso/iso ratio increased at low temperatures, leading to a concomitant increase in membrane fluidity (Beranova et al., 2010). More generally, even under aerobic conditions, in the presence of isoleucine, the expression of the FA desaturase gene *des* decreased at optimal and cold temperatures in *B. subtilis* (Cybulski et al., 2002). It is likely that the main mechanism utilized in *B. subtilis* to maintain membrane fluidity homeostasis is the synthesis of anteiso branched FAs. This does not appear to be the case for *B. cereus*, in which low temperature induces an increase in UFAs (mostly in the Δ5 position) but not in anteiso branched-chain FAs, even in media containing isoleucine (Brillard et al., 2010; De Sarrau et al., 2012).

In *B. cereus*, the i15/13 ratio decreased markedly at low temperatures (Diomandé et al., 2015b). In a lipase mutant of *B. cereus*, in which the i15/i13 ratio was much higher than that in the parental strain, growth impairment at low temperature was observed, emphasizing the probable role of a low i15/i13 ratio in psychrotolerance ability (Brillard et al., 2010). This result is consistent with the observation that this ratio is lower in psychrotrophic strains of *B. cereus sl.* (see the specific case of *Bacillus cereus sensu lato*). Such changes in the i15/i13 ratio have not been reported for other *Bacillus* species.

**FIGURE 6:**
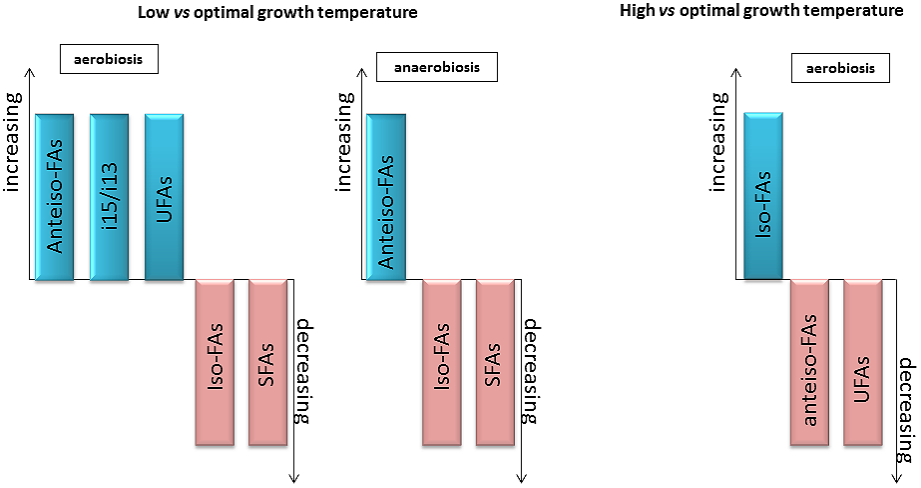
**General changes in FA composition observed with variations in temperature or O_2_ availability.** Increase (blue) or decrease (pink) of the type of varying FA in the condition considered.

In conclusion, the main changes observed in the FA composition of the genus *Bacillus* at low and high temperatures are presented in **Figure 6**. For low temperature adaptation, anteiso-, unsaturated FAs and i13 proportions increased, while iso-, saturated FAs and i15 proportions decreased. In contrast, the proportion of saturated FAs increased, and the proportions of anteiso and unsaturated FAs decreased at high temperature. The respective importance of these mechanisms may change with the species considered and the growth conditions.

### Changes Induced by other Conditions

Several other abiotic factors are known to induce modifications in the FA composition of *Bacillus* species.

When comparing the FA profile of *B. alkaliphilic* grown at pH 10, the proportion of UFAs and the anteiso/iso branched-chain FA ratio were lower when compared with the species grown at pH 7 (Yumoto et al., 2000). Similarly, for *B. subtilis* cells grown at pH 8.5 (Petrackova et al., 2010), the anteiso/iso branched-chain FA ratio and the total proportion of branched-chain FAs decreased compared with pH 7. These authors found the same trend, but with wider variations in cells grown at pH 5 compared with those grown at pH 7, resulting in more rigid membranes. In six different species of *Bacillus*, survival at a low pH (from 5 to 2) induced a global decrease in odd FAs, which are mostly branched-chain FAs in *Bacillus* (Shobharani and Halami, 2014), indicating that this may be a general adaptation to low pH among *Bacillus*. Moreover, a transcriptomic analysis during sorbic acid stress in *B. subtilis* revealed that genes important for FA biosynthesis were up-regulated, supporting the occurrence of plasma membrane remodeling in the stressed cells (Ter Beek et al., 2008). It was difficult to determine the FA modifications induced in *B. cereus* by reduced water activity because they depended on the solute used (sodium chloride versus sucrose) as well as on the strain (Haque and Russell, 2004).

Gamma irradiation treatment also induces changes in FA composition in some *Bacillus* species. In *B. cereus*, this treatment increases the proportion of UFAs (Ayari et al., 2009).

Low pressure is another influential factor: *B. subtilis* cells grown at 50 versus 1013 mbar show an increase in the ratio of unsaturated to saturated FAs but a decrease in the ratio of anteiso- to iso-FAs (Fajardo-Cavazos et al., 2012).

Some nanoparticles may be toxic for bacteria (Zhu et al., 2014). When *B. subtilis* was exposed to carbon nanotubes, an increased proportion of branched-chain FAs and a decreased amount of straight-chain FAs were observed (Zhu et al., 2014).

Thus, when facing various stressful environmental conditions such as those described above, *Bacillus* species alter their FA composition by increasing or decreasing the proportion of FAs with lower melting points, depending on the stress applied.

### Production of Bioactive FA-Derivatives

Under specific conditions, such as in response to the presence of some fungi (Li et al., 2014), colonization of the rhizosphere (Nihorimbere et al., 2009), biofilm formation (Hofemeister et al., 2004), or spreading to colonize a substrate (Angelini et al., 2009), some *Bacillus* species synthesize special FAs that are not integrated in their membrane but are components of excreted lipopeptides. These molecules display a wide range of bioactive properties, such as active surface properties and biocide capacity, and they play a key role in the adaptation of this *Bacillus* to various environments. For example, surfactin is a cyclic lipoheptapeptide that contains a β-hydroxy FA but no di-sulfide bridges or sugar residues. This molecule, which is produced by *B. subtilis subsp. subtilis* and *B. licheniformis*, possesses antimicrobial properties (Compaore et al., 2013).

*Bacillus amyloliquefaciens* is able to synthesize 26 types of surfactin, which act as potential antifungal metabolites. Among these, several new cyclic as well as acyclic surfactin variants have been identified based on the variation in the β-hydroxy fatty acid (β-OH FA) chain length and/or in amino acid positions 4, 5, 6, and 7 (Pathak et al., 2014). Other surfactins, such as those with long FA chains (C14 and C15) and characterized in *B. subtilis* (S499 strain), have insecticide effects on the fruit fly *Drosophila melanogaster* (Assie et al., 2002).

### Changes Depending on the Bacterial Cell State

The sporulation and germination of *Bacillus* species may also induce changes in the FA pattern. Indeed, it has been shown that *de novo* FA synthesis is required to establish cell type-specific gene transcription during sporulation in *B. subtilis* (Schujman et al., 1998).

In a study of ∼50 different species of *Bacillus* genus, the FA content of spores and cells were very similar (Song et al., 2000), but the proportion of branched-chain FAs (and more specifically, i15:0, the a15:0 and i17:0) was elevated in spores compared with vegetative cells. Moreover, some FAs present in vegetative cells were only detected at trace levels in spores (e.g., 3OH-C14:0 acid, iso-C16:1ω7c and anteiso-C17:1ω5c).

In *B. megaterium*, sporulation induced the synthesis of i14:0 branched-chain FAs in addition to the major FA i15:0 and, consequently, an increase in the proportion of branched-chain FAs was observed in the spores (Scandella and Kornberg, 1969). When the spores germinated, the proportion of branched-chain FAs returned to that observed in vegetative cells (Scandella and Kornberg, 1969).

In *B. weihenstephanensis*, spores obtained at 30°C displayed almost the same profile as vegetative cells, but during sporulation at 10°C, they displayed a different pattern (Planchon et al., 2011). In particular, the cold induced an increase in anteiso-branched-chain FAs but not UFAs, as observed in vegetative cells.

## Exogenous FAs: their Impact on *Bacillus* Growth

Fatty acids can be used as antimicrobials as a result of two main particularities: their lipophilicity and their acidity. To better understand the anti-microbial ability of exogenous FAs, several investigations have been performed, mostly in the food industry, which often aims to produce minimally processed food that is preserved against pathogens. Some examples are discussed below.

### Inhibition of Spore Germination

An antimicrobial activity of exogenous FAs resides in their capacity to inhibit spore germination and/or outgrowth. Indeed, in the presence of lauric acid (12C) and oleic acid (18C), *B. cereus* spore germination is completely inhibited (Ababouch et al., 1994). The authors hypothesized that this phenomenon resulted from the inhibition of germinant binding to germination sites. This inhibition was reversible because no additional inhibition was observed when the medium was depleted of these FAs.

Moreover, even in fully germinated spores, an inhibition of outgrowth was observed in the presence of lauric acid and two polyunsaturated FAs with 18C: linoleic and linolenic acids (Ababouch et al., 1994). The underlying mechanisms depended on the type of FAs, but the inner membrane of the germinated spores was shown to be the site of action of these inhibitors. Sorbic acid, a short UFA, has also been shown to delay the germination and outgrowth of *B. cereus* spores (Van Melis et al., 2011) as well as to inhibit spore germination (Smoot and Pierson, 1981).

Some FAs, such as laurate, palmitate, stearate and/or some FA esters, inhibit *Bacillus* spores in various culture conditions characterized by different temperatures, pressure and in combination with other chemical compounds such as sucrose or monoglycerol (Feijoo et al., 1997; Shearer et al., 2000; Klangpetch et al., 2013). However, for most of the cases cited above, an inhibitory rather than a lethal effect on the *Bacillus* spores was observed.

However, studies have also described the FAs, more specifically the UFAs, in terms of their ability to markedly decrease bacterial spore heat resistance, leading to a lethal effect on spores. A model has been used to describe the decrease in D-values (time for a log_10_ population decrease) in the presence of free FAs during low sterilization treatment (Lekogo et al., 2010).

### Growth Inhibition

Exogenous FAs may also inhibit the growth of vegetative cells. Linolenic acid, free or in addition to monoglyceride, displays strong antimicrobial activity against *B. cereus* cells (Lee et al., 2002). The addition of linolenic acid to medium was accompanied by a drastic increase in the bacterial extracellular ATP concentration and a decrease in the intracellular ATP concentration (Lee et al., 2002). Palmitic and stearic acid from clove oil also inhibited the growth of a range of microorganisms including *B. subtilis* (Assiri and Hassanien, 2013).

The presence of oleic acid markedly reduced the resistance of the protonophore-resistant strain C8 of *B. megaterium* to low concentrations of the carbonylcyanide m-chlorophenylhydrazone (CCCP) protonophore (Clejan et al., 1988). This loss of resistance was explained by the higher level of UFAs in the membrane of this bacterium when grown in presence of oleic acid. Conversely, the growth of the CCCP-sensitive wild-type strain in the presence of a saturated FA (stearic acid) improved resistance against the protonophore by increasing the saturated/unsaturated FA ratio (Clejan et al., 1988).

Thus, the effect of a given FA on growth appears to depend on both its properties and the environmental conditions.

### Improved Growth

In contrast, exogenous FAs may function as growth activators for vegetative *Bacillus* cells under particular conditions. As described above, *B. megaterium* can incorporate free FAs from the medium, and a modulation of resistance to the protonophores may be observed depending on the FA. Stearic acid (Clejan et al., 1988) in *B. megaterium* and palmitic acid in *B. subtilis* (Krulwich et al., 1987) are saturated FAs that are able to improve resistance against the protonophore.

*Bacillus cereus* can use exogenous phospholipids and integrate the FAs present in these phospholipids into its membrane (De Sarrau et al., 2013). For example, at low temperatures under anaerobiosis, the growth of *B. cereus* strains is presumably inhibited because membrane lipids and fluidity cannot adapt to low temperatures without oxygen (De Sarrau et al., 2012). UFAs from food (spinach) or from the bacterial medium can be incorporated into the membrane to facilitate the adaptation of strains grown at low temperature under anaerobiosis. Indeed, under this condition and in the presence of UFA precursors, the growth of these strains is similar to that under aerobiosis at low temperatures (De Sarrau et al., 2013).

In *B. cereus*, an exogenous source of UFAs was shown to improve the growth of the *casK/R* mutant (Diomandé et al., 2015a). This mutant displayed a decreased proportion of UFAs during growth at low temperatures compared to the parental strain, and an exogenous source of UFAs provided support to membrane-level modifications caused by the mutation.

Finally, some species, such as *B. megaterium*, may grow in medium supplemented with exogenous FAs, oxidize them and produce polyunsaturated FAs that are important industrial materials for the manufacture of valuable products such as oxygenated oils (Hou, 2009).

## Conclusion

Fatty acids are a universal pillar component of cellular membranes. FAs have been studied extensively in *Bacillus* species, and their involvement in a wide variety of adaptations highlights their predominant role in survival, growth and spore formation. In *Bacillus* species, FAs represent a good biomarker to determine the exact repartition among *Bacillus* species, depending on their environmental niche. At the metabolic level, FAS II is connected to major cell metabolic pathways, and FAs synthesis appears to be finely regulated depending on the needs of the cell. Numerous regulators control FA metabolism, and the regulation is clearly dependent on the environmental conditions of the cell.

**FIGURE 7:**
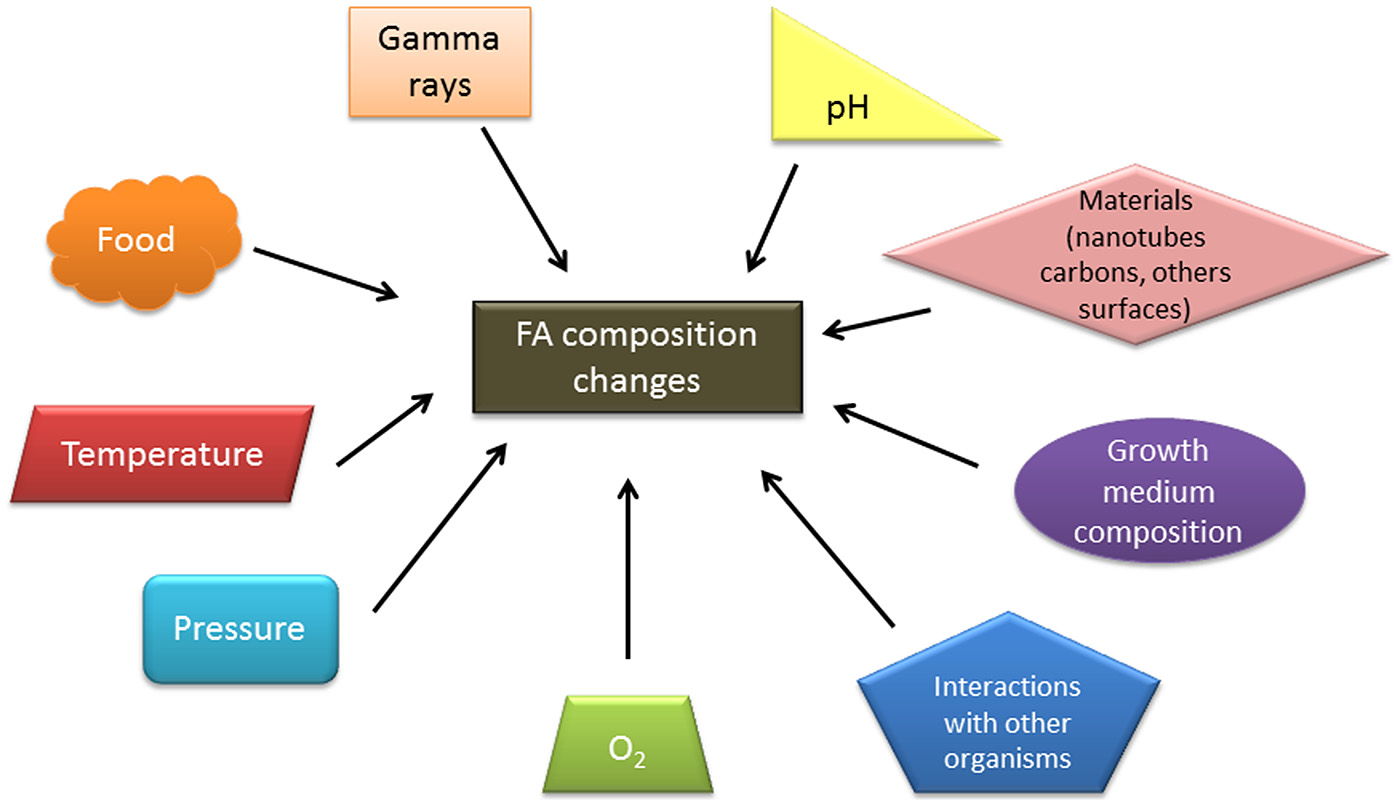
**Environmental factors influencing the FA composition of *Bacillus* genus strains**.

Depending on the environment, *Bacillus* species can display different FA patterns, which are mainly related to adaptation and survival (**Figure 7**). This ability to easily change their FAs profiles may contribute to the ubiquity of these species. Even if the FA composition varies in response to environmental fluctuations, some features of the FA pattern are conserved, which sometimes correlates to the growth ability of the species in a given environmental niche. Therefore, it is important to distinguish the short-term (short-time adaptation) from the long-term (long-term adaptation) change in FA composition during the evolution of a species. The impact of the observed changes in FA composition during the short-term adaptation of a species with different ecological abilities on membranes properties (e.g., fluidity, permeability) has seldom been measured, limiting the interpretation of their importance. There are some common features between the short-term and long-term adaptation of a species to temperature. In species grown at their lowest growth temperature domain versus their optimal temperature, and also in cold-adapted species grown at an optimal temperature versus mesophilic or thermotolerant species, a higher proportion of some UFAs and/or a higher ratio of anteiso/iso branched-chain FAs has been observed; both changes increase the membrane fluidity. These two main mechanisms do not seem to occur in *B. cereus* species, which display no clear changes in the anteiso/iso ratio, either during short-term adaptation to changes in temperature or long-term evolution of the phylogenetic groups within *B. cereus*. The impact of other changes on the membrane properties in relation to long-term and short-term adaptation to temperature, such as the i13/i15 ratio in *B. cereus*, remains unknown. It is possible that this change in the chain length of major FAs modifies the membrane fluidity. In contrast to temperature, there is no clear understanding of the role of FAs in the short-term and long-term adaptation to other environmental conditions (e.g., water activity, pH).

Some precautions should be taken because changes in FAs during short-term adaptation may lead to biases during the identification of strains. Therefore, it is important to combine genetic comparisons with the FA pattern and define optimal growth parameters for the newly described strain before assigning the genus and/or species affiliation.

*Bacillus* strains are also able to use FAs from their environment, including from food. This phenomenon is consistent with the observation that some *Bacillus* strains represent a major cause of foodborne illness. However, the impact of the use of these exogenous FAs may be positive or negative depending on the *Bacillus* cell state (spores or vegetative cells), the source of the FAs (e.g., free FAs or lipids), and/or the type of FAs, and it appears to depend on the environmental conditions. The antimicrobial activity of some of these exogenous FAs might be effective for food preservation during processing and for the conservation of food.

Altogether, the relationship between the bacterial FA composition and the physiological impact under environmental stresses might be the timely suitable and important issue to be further investigated in detail.

## Author Contributions

SD analyzed the data from the literature and organized and wrote the manuscript. CN-T analyzed the data and was involved in writing the final version of the manuscript. M-HG and VB analyzed the data and edited the manuscript. JB analyzed the data and was involved in writing the different versions of the manuscript. All of the authors read and approved the final version of the manuscript.

## Conflict of Interest Statement

The authors declare that the research was conducted in the absence of any commercial or financial relationships that could be construed as a potential conflict of interest.
